# Establishment and validation of prognostic nomograms in female lung adenocarcinoma patients

**DOI:** 10.1097/MD.0000000000045170

**Published:** 2025-10-17

**Authors:** Feiyang Li, Fang Li, Dong Zhao, Haowei Lu

**Affiliations:** a Ward 2, Department of Medical Oncology, Lixin People’s Hospital of Bozhou City, Anhui Province, China; b Ward 1, Department of Medical Oncology, Affiliated Hospital of Qinghai University, Lixin, Anhui Province, China.

**Keywords:** lung adenocarcinoma, nomograms, predictive modeling, SEER database, women

## Abstract

The incidence of lung adenocarcinoma in women is gradually increasing, but the prognostic factors affecting this group of patients have not been systematically studied, so we hope that we can construct a prognostic prediction model for this group of patients to provide a more accurate survival prediction. We performed a retrospective analysis of female patients with pathologically diagnosed lung adenocarcinoma, constructed nomograms of overall survival (OS) and cancer-specific survival (CSS) at 1, 3, and 5 years using COX regression analyses, and evaluated the prediction using the consistency index (C-index), calibration curves, receiver operating characteristic (ROC) curves, and decision curve analysis (DCA) model performance with internal and external validation. We included a total of 11,562 patients, which were divided into 2 groups in a ratio of 7:3 Analysis using the chi-square test revealed that there was no statistically significant difference in the baseline information between the 2 data groups (*P* > .05). Age, race, marital status, AJCC stage, surgery, radiotherapy, chemotherapy, and distant metastasis were found to be influential factors for OS using COX regression analysis, and we used these influences to construct prognostic nomograms for OS. The same method was then used to screen the independent prognostic influences affecting CSS were age, marital status, AJCC stage, surgery, radiotherapy, chemotherapy, and distant metastasis, and prognostic nomograms for CSS were constructed using these factors. The prognostic models for OS and CSS were validated using ROC curves, C-indexes, correction curves, and DCA curves after the construction was completed, proving the accuracy and reliability of our models. This prediction model can more accurately predict the prognosis of female lung adenocarcinoma patients.

## 1. Introduction

Lung cancer, as one of the malignant tumors with the highest morbidity and mortality rates worldwide, poses a serious threat to human health, and according to the global cancer statistics for 2022 there are approximately 2.5 million new lung cancer cases and 1.796 million deaths globally, accounting for 12.4% and 18.7% of all malignant tumors, respectively.^[1]^ And the incidence of lung cancer is still on the rise for some time to come, according to a study conducted by Rajesh Sharma et al^[2]^ by 2050, it is expected that there will be 3.8 million new cases of lung cancer and 3.2 million deaths globally, thus lung cancer will remain a malignancy of great concern for some time to come. In developed countries, such as the United States, the morbidity and mortality rates of lung cancer have been stabilized through the implementation of tobacco control measures, while in developing countries, the morbidity and mortality rates of lung cancer are still on the rise, and with about 871,000 new cases of lung cancer and 767,000 deaths in China in 2022, the prevention and treatment of lung cancer are still faced with great challenges.^[3,4]^

Constant segmentation of lung cancer patients and precise personalized treatment for specific subgroups of patients is an important way forward in lung cancer research, and many studies have been conducted on lung cancers of different genders, races, age groups, and pathological subtypes, especially with regard to gender, both in terms of causes, incidence, treatment, and prognosis for male and female lung cancers.^[5]^ Lung cancer was previously thought to be a male disease with smoking as the main cause, but some studies in recent years have found that although the proportion of female smokers is lower, women are more susceptible to tobacco carcinogenesis because of sex differences in the levels of the cytochrome P450 enzyme, CYP1A1, in addition to household coal burning and cooking with solid fuels, which may also be one of the etiologic factors for lung cancer in women.^[6,7]^ In terms of incidence, according to GLOBOCAN 2020, the incidence of female lung cancer patients is gradually increasing, especially in North America and Oceania, where the incidence of lung cancer is higher in women than in men.^[4]^ Also in terms of pathologic type, the most common pathologic type is adenocarcinoma because there are more nonsmoking patients in women, and the mutation rate of driver genes is also higher in female patients, and these targets are important for the treatment of lung cancer.^[8]^ Female lung cancer patients show better response to chemotherapy and immunotherapy due to differences in sex hormone levels and immune response, which may also be the reason why the prognosis of female patients is better than that of male patients.^[9,10]^

From the above studies, we can find that female lung cancer patients have unique characteristics and prognosis compared with male patients, and the incidence rate of female lung cancer patients is gradually increasing, but this group of female lung cancer patients has not been paid enough attention. For example, an analysis of 426 phase III clinical trials in lung cancer conducted between 1984 and 2019 found that the percentage of female patients included was 30.98%, just half that of male patients.^[11]^ Moreover, we searched PubMed and found that there is no systematic study on the prognostic factors of female lung adenocarcinoma. Therefore, it is for the above reasons that the authors hope to use the SEER database and external cohorts of female lung adenocarcinoma patients to construct a prognostic prediction model that is accurate, efficient, and easy to use, so as to help clinicians to more accurately predict the prognosis of this group of patients and provide a basis for the individualization of the treatment and management of patients. This will provide a basis for the individualized treatment and management of patients and provide a reference for the formulation of guidelines related to female lung adenocarcinoma.

## 2. Materials and methods

### 2.1. Data sources and research design

We used SEER*Stat 8.4.3 software to obtain data on patients with pulmonary malignancies from the April 17, 2024 release, which included tumor stage, clinicopathologic, and survival information. Prior to commencing this study, we submitted a data use agreement to the SEER project team and were formally granted access to the database under approval number 14038-Nov2021. SEER is a publicly accessible research database, and the personal information within the database has been de-identified. Therefore, this study did not require ethical approval or informed consent. The study was conducted in accordance with the Declaration of Helsinki (revised 2013). These data were screened by inclusion exclusion criteria, which were as follows: year of diagnosis 2010 to 2015; gender female; and ICD-O-3 pathologic diagnosis: 8140/3: Adenocarcinoma, NOS. The exclusion criteria were as follows: patients with survival < 1 month; and patients with missing key information.

### 2.2. Research variables

In constructing the predictive model, we initially selected variables based on potential influencing factors identified in prior studies. These factors include various aspects, such as demographic characteristics, lifestyle factors, clinical manifestations, and treatment-related factors. Previous studies provided valuable insights into prognostic factors for lung cancer patients. Building on these identified factors, we incorporated clinical experience and exploratory data analysis to preliminarily identify variables that could significantly impact the prognosis of female lung adenocarcinoma patients, including age, race, marital status, AJCC stage, surgery, radiotherapy, chemotherapy, and distant metastasis.^[12–15]^ The selection of these variables aims to ensure comprehensive coverage of the key factors that may influence the prognosis of female lung adenocarcinoma patients, thus providing a strong foundation for subsequent multivariate analysis and the development of the predictive model. First, in accordance with the established inclusion and exclusion criteria, we screened the patient data and split the filtered dataset into training and validation sets in a 7:3 ratio, from which relevant variables were extracted. The demographic characteristics data included age (18–59.9 years, 60–79.9 years, 80+ years), race (black, white, other race), and marital status (divorced, married, and single). Tumor characteristics included tumor location (left lung, right lung), AJCC stage (stage I, II, III, and IV), surgery (yes or no), radiation therapy (yes or no), chemotherapy (yes or no), and information on distant metastasis (bone metastasis, brain metastasis, liver metastasis, and lung metastasis). There is also information on the patient’s survival time, survival status and cause of death.

### 2.3. Statistical analysis

The demographic characteristics information and tumor-related information of the training and validation sets were compared using the chi-square test; the prognostic influences of overall survival (OS) and cancer-specific survival (CSS) were screened by COX unifactorial and multifactorial screening, and the prognostic prediction models were constructed using these factors and nomograms were plotted, which were used to predict the 1-, 3-, and 5-year survival rates of OS and CSS. We used consistency index (C-index) to assess model discrimination, and C-index thresholds for nomograms prediction accuracy were defined as low (0.50–0.70), medium (0.71–0.90), and high accuracy (>0.90).^[16]^ The model was evaluated using the receiver operating characteristic (ROC) curve and the area under the curve (AUC), with an AUC of 0.50 to 0.70 being considered less accurate, between 0.71 to 0.90 being moderately accurate, and above 0.90 being highly accurate.^[17]^ The calibration of the model was tested by plotting a calibration curve using bootstrap method with 1000 replicate samples to ensure the accuracy of the model. Decision curve analysis (DCA) curves were plotted for the model to test the clinical benefit and application value of the model. We use validation sets for internal validation of the constructed models and external queues for external validation. Finally, we stratified the risk according to the nomograms score, and after stratification, we compared the survival time of the 2 groups using Kaplan–Meier analysis. All statistical analyses were performed using R software (version 4.4.1) and were statistically significant at *P* < .05.

## 3. Results

### 3.1. Patient characteristics

**Figure 1.**
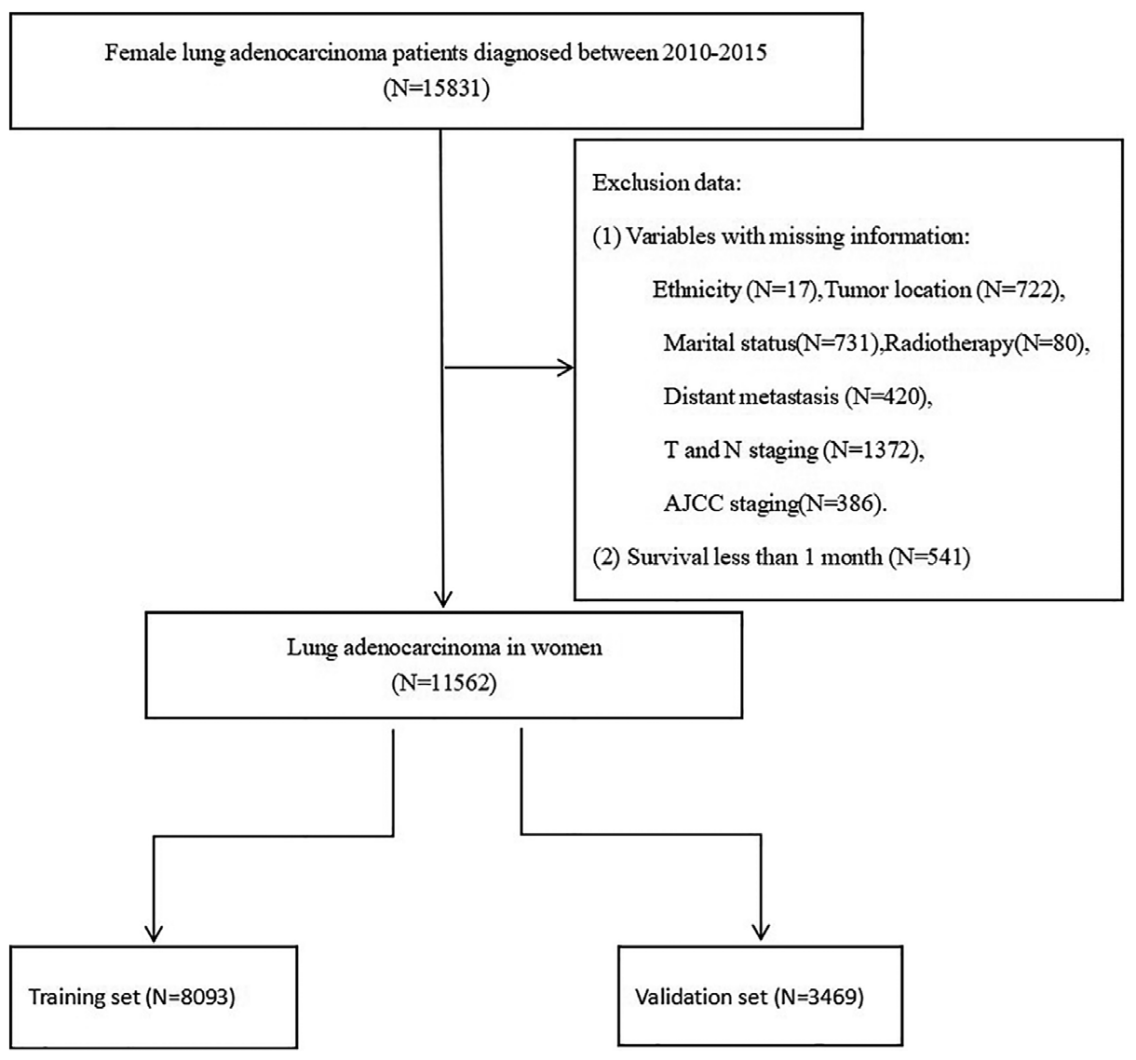
Workflow diagram.

In this study, a total of 15,831 female lung adenocarcinoma patients were screened from the SEER database, and those who met the inclusion criteria and did not meet the exclusion criteria were 11,562, which were randomized into a training set (N = 8093) and a validation set (N = 3469) according to a 7:3 randomization (Fig. 1).The age in both the training and validation sets was mainly 60 to 79.9 years, accounting for about 61% or so of all patients; the proportion of Caucasians among patients reached more than 78% in both cases, blacks accounted for about 8% or so of all patients, and the ratio of other races was about 12%; and with regard to the marital status, the majority of the patients were married at the time of diagnosis, with a proportion of about 45%. The percentage of patients with stage IV in AJCC staging was about 44%, followed by stage I patients with about 30%, which is consistent with some previous reports of lung adenocarcinomas in women, where the percentage of patients with stage I was higher than that of other subgroups of lung cancer. In terms of treatment, the proportion of surgical patients was 30%, the proportion of patients who underwent radiotherapy radiation therapy was about 43%, while the ratio of systemic chemotherapy was about 48%. In terms of distant metastasis, the proportion of bone metastasis was still the highest, about 17%, followed by brain metastasis and lung metastasis, about 13%, and liver metastasis was the least common, with the proportion of 6%; in addition, the baseline data of the patients in both groups of the training and validation sets were similar, with no statistical difference (*P* > .05) (Table 1).

**Table 1:** Baseline patient information.

Variable	Total (n = 11,562)	Training (n = 8093)	Validation (n = 3469)	χ^2^	*P*
Age, n (%)					
18–59.9 yr	2337 (20.21)	708 (20.41)	1629 (20.13)	0.58	.746
60–79.9 yr	7118 (61.56)	2143 (61.78)	4975 (61.47)		
80+ yr	2107 (18.22)	618 (17.81)	1489 (18.40)		
Race, n (%)					
Black	988 (8.55)	307 (8.85)	681 (8.41)	0.61	.736
Other	1493 (12.91)	449 (12.94)	1044 (12.90)		
White	9081 (78.54)	2713 (78.21)	6368 (78.69)		
Marital status, n (%)					
Married	5306 (45.89)	1611 (46.44)	3695 (45.66)	1.77	.412
Single	3406 (29.46)	992 (28.60)	2414 (29.83)		
Widowed	2850 (24.65)	866 (24.96)	1984 (24.52)		
Laterality, n (%)					
Left	4739 (40.99)	1432 (41.28)	3307 (40.86)	0.17	.676
Right	6823 (59.01)	2037 (58.72)	4786 (59.14)		
AJCC stage group, n (%)					
I	3491 (30.19)	1048 (30.21)	2443 (30.19)	1.38	.710
II	900 (7.78)	255 (7.35)	645 (7.97)		
III	1999 (17.29)	600 (17.30)	1399 (17.29)		
IV	5172 (44.73)	1566 (45.14)	3606 (44.56)		
Surgery, n (%)					
No	8051 (69.63)	2406 (69.36)	5645 (69.75)	0.18	.673
Yes	3511 (30.37)	1063 (30.64)	2448 (30.25)		
Radiation, n (%)					
No	6557 (56.71)	2001 (57.68)	4556 (56.30)	1.90	.168
Yes	5005 (43.29)	1468 (42.32)	3537 (43.70)		
Chemotherapy, n (%)					
No	5934 (51.32)	1793 (51.69)	4141 (51.17)	0.26	.609
Yes	5628 (48.68)	1676 (48.31)	3952 (48.83)		
Bone metastasis, n (%)					
No	9492 (82.10)	2843 (81.95)	6649 (82.16)	0.07	.794
Yes	2070 (17.90)	626 (18.05)	1444 (17.84)		
Brain metastasis, n (%)					
No	9949 (86.05)	2983 (85.99)	6966 (86.07)	0.01	.905
Yes	1613 (13.95)	486 (14.01)	1127 (13.93)		
Liver metastasis, n (%)					
No	10,831 (93.68)	3239 (93.37)	7592 (93.81)	0.79	.373
Yes	731 (6.32)	230 (6.63)	501 (6.19)		
Lung metastasis, n (%)					
No	9959 (86.14)	2985 (86.05)	6974 (86.17)	0.03	.858
Yes	1603 (13.86)	484 (13.95)	1119 (13.83)		

a = American Indian/AK Native, Asian/Pacific Islander.

### 3.2. Independent prognostic factors for OS and CSS

One-way COX regression analysis of variables that may affect OS in the training set showed that age, race, marital status, AJCC stage, surgery, radiotherapy, chemotherapy, and distant metastasis (bone, brain, liver, and lung) were prognostic influencers of OS (*P* < .1), and multifactorial COX regression analysis of these variables found that age, race, marital status, AJCC stage, surgery, radiotherapy, chemotherapy and distant metastasis (bone, brain, liver) were independent prognostic influences on OS (*P* < .05) (Table 2). We used the same method to screen the independent prognostic factors affecting CSS, including: age, marital status, AJCC stage, surgery, chemotherapy and distant metastasis (bone, brain and liver) (*P* < .05) (Table 3).

**Table 2:** Univariate and multivariate analyses of OS in training set.

Variate	Univariate analysis			Multivariate analysis		
	*P*	HR	95% CI	*P*	HR	95% CI
Age						
18–59.9 yr	Reference			Reference		
60–79.9 yr	.140	1.05	0.98–1.12	<.001	1.19	1.11–1.28
80+ yr	<.001	1.56	1.44–1.69	<.001	1.61	1.47–1.76
Race						
Black	Reference			Reference		
Other	.036	0.89	0.79–0.99	<.001	0.69	0.62–0.78
White	.273	0.95	0.87–1.04	.044	0.91	0.83–0.99
Marital status						
Married	Reference			Reference		
Single	<.001	1.12	1.05–1.19	<.001	1.12	1.05–1.19
Widowed	<.001	1.31	1.23–1.39	.003	1.11	1.04–1.19
Laterality						
Left	Reference			–	–	–
Right	.085	0.96	0.91–1.01	–	–	–
AJCC stage group						
I	Reference			Reference		
II	<.001	1.68	1.50–1.87	<.001	2.02	1.80–2.26
III	<.001	2.51	2.31–2.73	<.001	2.95	2.69–3.24
IV	<.001	5.37	5.01–5.75	<.001	4.42	4.00–4.89
Surgery						
No	Reference			Reference		
Yes	<.001	0.22	0.21–0.24	<.001	0.39	0.35–0.42
Radiation						
No	Reference			Reference		
Yes	<.001	1.53	1.45–1.61	.013	0.93	0.87–0.98
Chemotherapy						
No	Reference			Reference		
Yes	<.001	1.52	1.44–1.60	<.001	0.55	0.52–0.59
Bone metastasis						
No	Reference			Reference		
Yes	<.001	2.80	2.63–2.98	<.001	1.33	1.24–1.43
Brain metastasis						
No	Reference			Reference		
Yes	<.001	2.42	2.26–2.59	<.001	1.29	1.19–1.40
Liver metastasis						
No	Reference			Reference		
Yes	<.001	3.04	2.76–3.34	<.001	1.49	1.35–1.64
Lung metastasis						
No	Reference			Reference		
Yes	<.001	2.31	2.16–2.47	.640	1.02	0.95–1.10

a = American Indian/AK Native, Asian/Pacific Islander, OS = overall survival.

**Table 3:** One-factor and multi-factor COX analysis of CSS in the training set.

Variate	Univariate analysis			Multivariate analysis		
	*P*	HR	95% CI	*P*	HR	95% CI
Age						
18–59.9 yr	Reference			Reference		
60–79.9 yr	.015	0.92	0.85–0.98	.490	1.03	0.95–1.10
80+ yr	.020	0.90	0.83–0.98	.048	1.11	1.01–1.22
Race						
Black	Reference			***–***	–	–
Other	.142	0.91	0.81–1.03	–	–	–
White	.341	0.95	0.86–1.05	***–***	–	–
Marital status						
Married	Reference			Reference		
Single	.045	1.07	1.01–1.14	.015	1.09	1.02–1.16
Widowed	.959	1.00	0.93–1.07	.147	1.06	0.98–1.14
Laterality						
Left	Reference			–	–	–
Right	.569	0.98	0.93–1.04	–	–	–
AJCC stage group						
I	Reference			Reference		
II	<.001	1.59	1.39–1.81	<.001	1.92	1.68–2.19
III	<.001	2.09	1.89–2.32	<.001	2.70	2.42–3.02
IV	<.001	4.04	3.71–4.41	<.001	4.21	3.75–4.72
Surgery						
No	Reference			Reference		
Yes	<.001	0.37	0.35–0.41	<.001	0.60	0.55–0.66
Radiation						
No	Reference			Reference		
Yes	<.001	1.14	1.07–1.20	.355	0.97	0.91–1.04
Chemotherapy						
No	Reference			Reference		
Yes	<.001	1.26	1.19–1.33	<.001	0.57	0.54–0.62
Bone metastasis						
No	Reference			Reference		
Yes	<.001	2.17	2.04–2.32	<.001	1.29	1.20–1.39
Brain metastasis						
No	Reference			Reference		
Yes	<.001	1.92	1.79–2.06	<.001	1.20	1.11–1.31
Liver metastasis						
No	Reference			Reference		
Yes	<.001	2.30	2.08–2.53	<.001	1.40	1.26–1.55
Lung metastasis						
No	Reference			Reference		
Yes	<.001	1.71	1.59–1.83	.366	0.97	0.89–1.04

a = American Indian/AK Native, Asian/Pacific Islander, CSS = cancer-specific survival.

### 3.3. Construction of nomograms for OS and CSS prognosis

**Figure 2.**
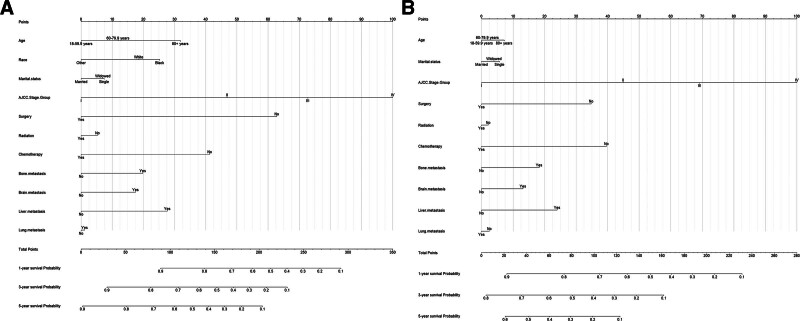
Prognostic nomograms of 1-, 3-, and 5-yr OS (A) and CSS (B). CSS = cancer-specific survival, OS = overall survival.

We constructed column-line plots on OS and CSS using the prognostic influences screened by COX regression analysis and predicted patient survival by integrating all predictors in the plotted nomograms. The total score obtained by summing the scores obtained for each variable predicted the 1-, 3-, and 5-year survival of patients (Fig. 2).

### 3.4. Validation of the nomograms graph

**Figure 3.**
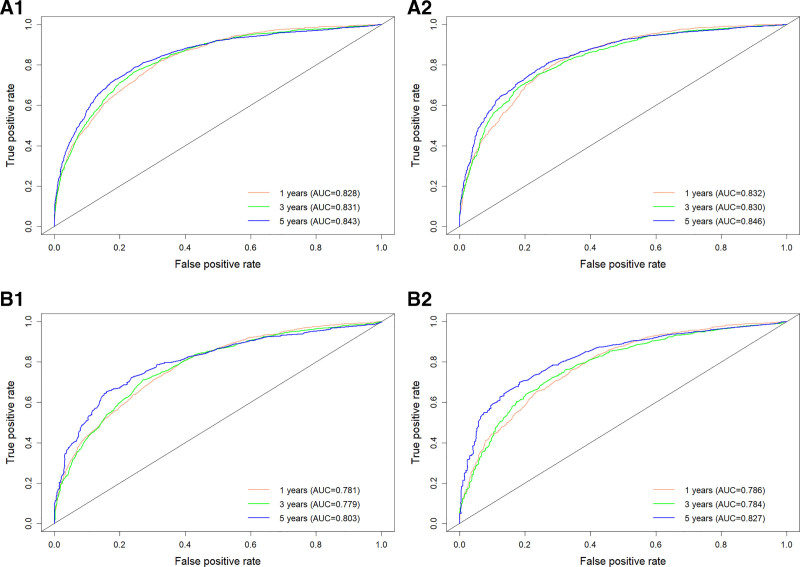
A1 is the OS training set ROC curve, A2 is the OS validation set ROC curve; B1 is the CSS training set ROC curve and B2 is the CSS validation set ROC curve. CSS = cancer-specific survival, OS = overall survival, ROC = receiver operating characteristic.

**Figure 4.**
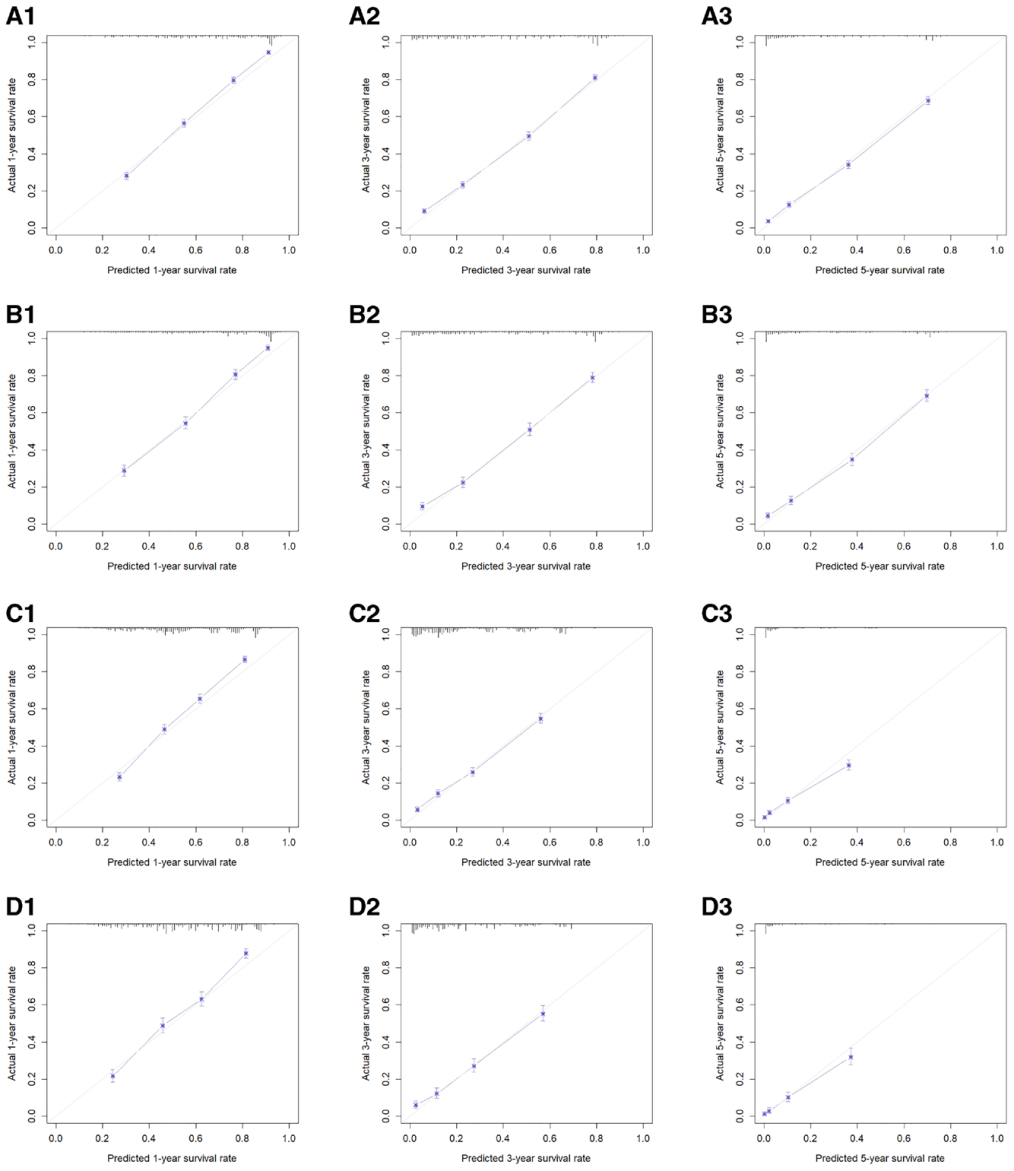
A1, A2 and A3 are 1-yr, 3-yr and 5-yr calibration curves of OS training set, respectively; B1, B2 and B3 are 1-year, 3-yr and 5-yr calibration curves of OS verification set, respectively. C1, C2 and C3 are the 1-yr, 3-yr and 5-yr calibration curves of the CSS training set, respectively; D1, D2 and D3 are the 1-yr, 3-yr and 5-yr calibration curves of the CSS verification set, respectively. CSS = cancer-specific survival, OS = overall survival.

By using the R software, the C-indexes were calculated to be 0.759 and 0.763 for the OS training and validation sets, respectively, and 0.719 and 0.727 for the CSS training and validation sets, respectively, indicating that this model has a good predictive value (Table 4). Risk scores for each of the independent prognostic factors were continued to be calculated using the R software and ROC curves for 1-, 3-, and 5-year survival were plotted for the training set for OS and CSS and validated using the validation set by plotting ROC curves for 1-, 3-, and 5-year survival. The 1-, 3-, and 5-year AUC values were 0.828, 0.831, and 0.843 for the OS training set, and 0.832, 0.830, and 0.846 for the 1-, 3-, and 5-year AUC values for the OS validation set; The 1-, 3-, and 5-year AUC values for the CSS training set were 0.781, 0.779, and 0.803, and the 1-, 3-, and 5-year AUC values for the CSS validation set were 0.786, 0.784, and 0.827 (Fig. 3). The above results show the ability of this prediction model to assess OS and CSS at 1, 3, and 5 years in female lung adenocarcinoma patients relatively reliably. This model was then validated using the bootstrap method, setting the sampling number *B* = 1000, and the validation results showed that the 1-, 3-, and 5-year survival correction curves for OS and CSS were similar to the 45° reference line, indicating a more accurate prediction (Fig. 4).

**Table 4:** C-index results.

	C-index	se(C-index)
OS		
Training set	0.759	0.003
Validation set	0.763	0.004
External validation set	0.709	0.032
CSS		
Training set	0.719	0.003
Validation set	0.727	0.005
External validation set	0.662	0.044

C-index = consistency index, CSS = cancer-specific survival, OS = overall survival.

### 3.5. DCA analysis

**Figure 5.**
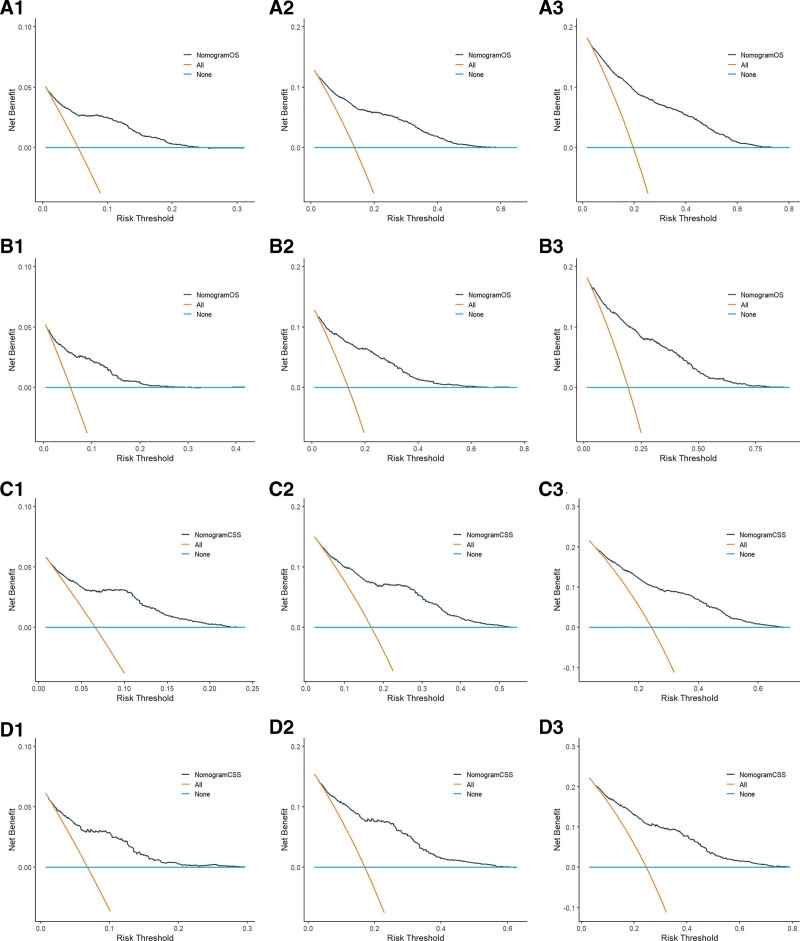
A1, A2, and A3 are the 1-yr, 3-yr, and 5-yr DCA curves for the OS training set; B1, B2, and B3 are the 1-yr, 3-yr, and 5-yr DCA curves for the OS validation set; C1, C2, and C3 are the 1-yr, 3-yr, and 5-yr DCA curves for the CSS training set; D1, D2, and D3 are the 1-yr, 3-yr, and 5-yr DCA curves for the CSS validation set, respectively. CSS = cancer-specific survival, DCA = decision curve analysis, OS = overall survival.

DCA is an assessment method used to evaluate the degree of patient benefit. The area under the ROC curve measures only the diagnostic accuracy of the predictive model and fails to take into account the clinical utility of a particular model, which may lead to overmedication, which can be well addressed by the DCA curve.^[18]^ In this study, DCA curves were plotted based on 1-, 3-, and 5-year survival of patients in OS and CSS prognostic models, respectively. The results showed higher 1-, 3-, and 5-year net clinical benefit in both models established in this study (Fig. 5).

### 3.6. Nomograms-based risk stratification

**Figure 6.**
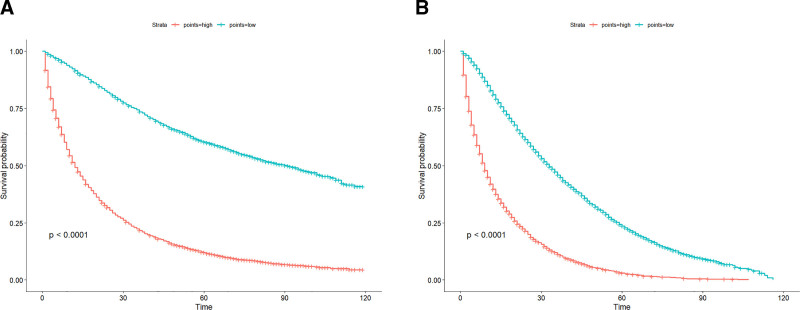
A graph shows the K–M survival curves of OS between the low-risk and high-risk groups; B graph shows the K–M survival curves of CSS between the low-risk and high-risk groups. CSS = cancer-specific survival, OS = overall survival.

Working with the nomogramFormula package, we calculated scores for each patient based on the constructed nomograms and categorized patients into 2 risk groups based on the scores: low-risk and high-risk groups. Kaplan–Meier OS curves and Kaplan–Meier CSS curves showed that patients in the low-risk group had significantly better survival times than those in the high-risk group and that there was a statistically significant difference between the 2 risk groups (*P* < .05) (Fig. 6).

### 3.7. External validation

**Figure 7.**
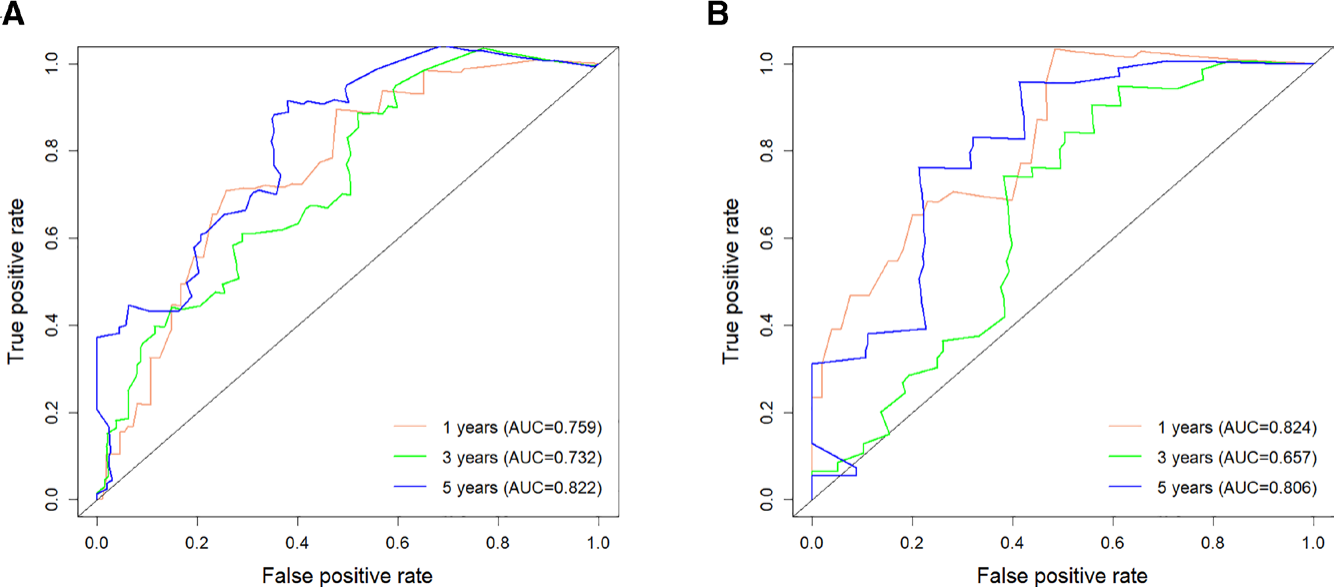
Graph A shows the ROC curve for the external validation set OS; Graph B shows the ROC curve for the external validation set CSS. CSS = cancer-specific survival, OS = overall survival, ROC = receiver operating characteristic.

**Figure 8.**
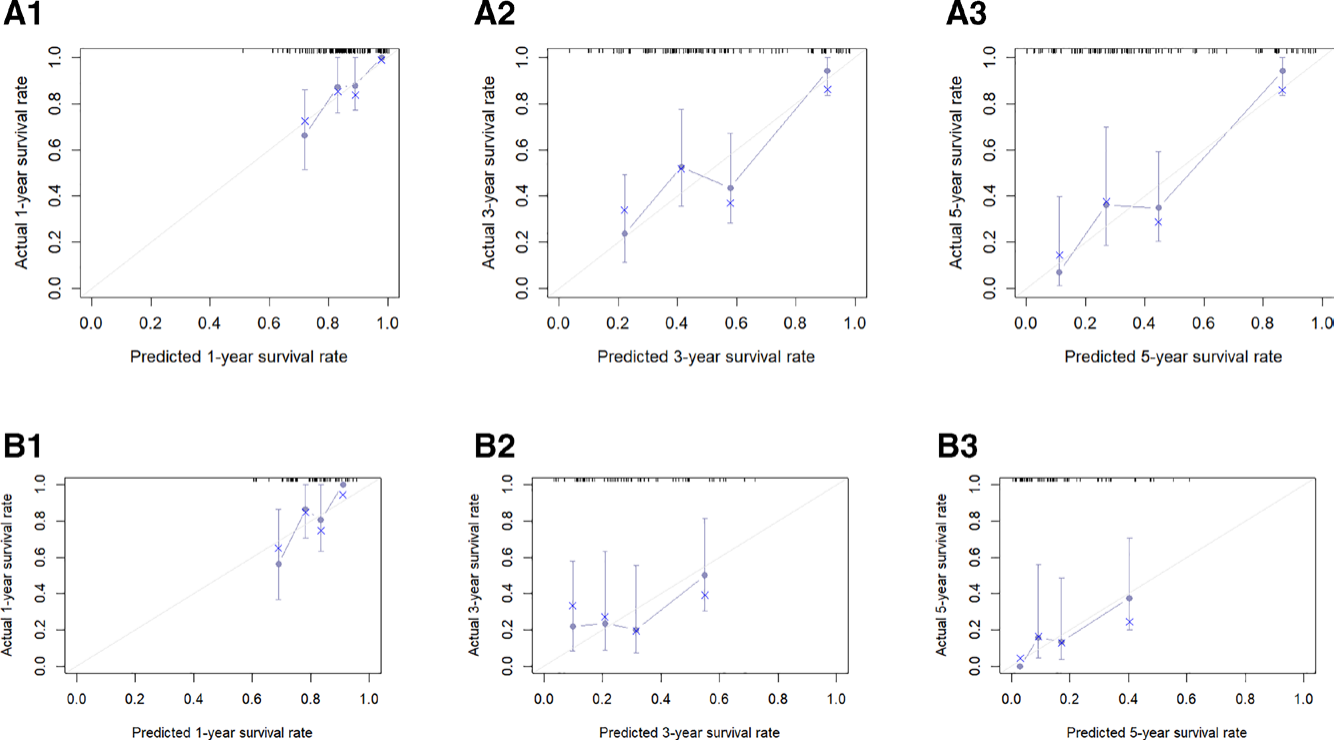
A1, A2, and A3 are the 1-, 3-, and 5-yr calibration curves for the external validation set OS; B1, B2, and B3 are the 1-, 3-, and 5-yr calibration curves for the external validation set CSS, respectively. CSS = cancer-specific survival, OS = overall survival.

**Figure 9.**
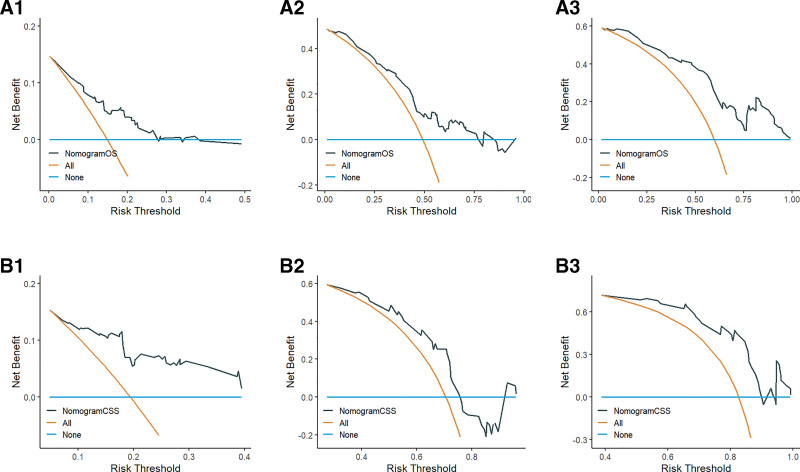
A1, A2, and A3 are the 1-, 3-, and 5-yr DCA curves for the external validation set OS; B1, B2, and B3 are the 1-, 3-, and 5-yr DCA curves for the external validation set CSS, respectively. CSS = cancer-specific survival, DCA = decision curve analysis, OS = overall survival.

We constructed an external cohort of 130 female lung adenocarcinoma patients collected at our center based on inclusion and exclusion criteria for validation. The final validation results showed that the C-indexes for OS and CSS were 0.709 and 0.662, respectively (Table 4);The 1-, 3-, and 5-year AUCs for OS were 0.759, 0.732, and 0.822, respectively, and the 1-, 3-, and 5-year AUCs for CSS were 0.824, 0.657, and 0.806, respectively (Fig. 7);The calibration curves of the model were validated using external data, and the 1-, 3-, and 5-year survival calibration curves for OS and CSS were similar to the 45° reference line (Fig. 8);In addition, DCA curves were plotted using external data and the final results showed higher net clinical benefit at 1, 3 and 5 years (Fig. 9).Through validation with external data, we demonstrate the feasibility of applying the model to different populations.

## 4. Discussion

With the implementation of tobacco control and other measures, the incidence and mortality rates of lung cancer are stabilizing, but the incidence of lung cancer in certain subgroups is still rising rapidly, especially in female nonsmokers. During the period of 2000 to 2016, the standardized incidence rate of lung cancer in Chinese males has increased by about 0.8% per year, while the annual growth rate in females has reached 2.1%, which is a very alarming rate of increase. And there is some difference between female lung cancer and male lung cancer.^[19]^ And there is no previous systematic study on the factors influencing the prognosis of women’s lung cancer, so we hope to construct a prognostic prediction model about women’s lung adenocarcinoma by using the data in the SEER database, which will provide some basis for clinical treatment and research. The SEER database is a large North American tumor registry database that covers approximately 30% of the North American population and can provide us with diverse and reliable data for this study.^[20]^ Finally, we used these data to successfully construct a prognostic prediction model about OS and CSS and plotted nomograms, using ROC curves, C-indexes, correction curves and DCA curves to prove the accuracy and practicality of the model, and we also used external cohorts to validate the feasibility of applying this model in different populations, and we also obtained better results.

Age is a very important prognostic influence in the model for both OS, and CSS, and with age, patients have more comorbid underlying diseases, decreased organ reserve capacity, and worse nutritional status, which contribute to increased mortality.^[21]^ In our study especially patients over 80 years of age increased the risk of death by 61%, while the effect on CSS was smaller, increasing the risk of death by only 11%, which may be related to the less malignant tumors and slower tumor progression in older patients, but overall age leads to a poorer prognosis, which is similar to a previous study by Chao Li et al who also found that age is positively correlated with mortality.^[22,23]^ Race aspect in our study showed that lung adenocarcinoma mortality was higher in female blacks compared to other races, but this effect was only associated with OS (HR = 0.69, 95% CI: 0.62–0.78). The reason for this may be due to the fact that blacks have higher non-tumor-related causes of death, which was also confirmed in a study by Xiao Wu^[24]^ and others, in which non-Hispanic blacks had a higher risk of cardiovascular disease mortality compared to non-Hispanic whites. The effect of marital status on lung adenocarcinoma mortality is undisputed, which has been supported in many previous studies, and the prognosis of married patients is better than that of unmarried or single individuals, probably because married patients are more able to receive psychological support from their families and are more likely to receive antitumor therapy, so increasing the survival rate of the patients.^[25,26]^

AJCC staging, which is formulated based on anatomical factors, is currently the most widely used and mature staging system in clinical practice, and it can provide a very important role in the development of treatment plans and prognosis.^[27]^ The prognostic impact of AJCC staging in our study was enormous, especially the exponentially increased risk of death in stage III and IV patients relative to stage I patients. However, AJCC staging has some drawbacks, such as its staging basis is mainly based on anatomical factors, and in recent years the staging basis has also referred to some biomarkers, but it ignores the impact of some common demographic characteristics on prognosis, and at the same time, this is also one of the reasons why we constructed this model.^[28]^ Currently, common treatments for malignant tumors include surgery, radiotherapy, chemotherapy, immunotherapy, and targeted therapy, but unfortunately, the SEER database only has information on surgery, radiotherapy, and chemotherapy. For early-stage lung adenocarcinoma, a favorable prognosis can be achieved with surgical treatment, and female patients with lung adenocarcinoma are more likely to benefit from surgery. A retrospective analysis included 735 surgically treated non-small cell lung cancers, including 246 female patients, and the final results showed that the 5-year overall survival rate was significantly higher in female patients than in males (76.2% vs 57.3%), which may be explained by a history of nonsmoking and a lower cardiovascular prevalence.^[29]^ Whereas male patients were not included in our study and therefore could not be compared to male patients, we also found that surgery was an independent prognostic influence on prognosis, reducing the risk of death by 61% compared to non-operated patients (HR = 0.39, 95% CI: 0.35–0.42). Radiotherapy is also one of the important treatments for malignant tumors, and in our study radiotherapy resulted in a benefit in OS but not in CSS, we speculate that this may be because the radiotherapy data included in the SEER database includes a portion of palliative radiotherapy, which does not affect the CSS of patients, but this is only a speculation, and needs to be confirmed by a study specifically for women with lung adenocarcinoma. In addition, chemotherapy had a significant benefit both for OS, and CSS, reducing the risk of death by about 40%. The superior efficacy of chemotherapy in female patients compared to male patients may be due to the difference in the immune microenvironment, with female lung cancer patients having higher levels of immune cell infiltration.^[30]^ A previous study from the Swedish Cancer Registry showed that the most common site of metastasis for lung adenocarcinoma was the skeleton, followed by the respiratory system, and female lung cancer patients were more likely to have neurological metastases, which is basically consistent with our study, and the study also showed that the OS time of metastatic lung cancer would be drastically shortened to only about 5 months, and in particular the survival of patients with hepatic metastases would only be about 3 months, and the average survival of patients with non-metastatic The average survival of lung cancer patients after diagnosis is 13 months, and the impact of distant metastasis on survival prognosis is huge, and the same conclusion was obtained in our study, so we included distant metastasis in this model.^[31]^

Nomograms are visual representations based on statistical models, commonly utilized in medical research and clinical practice, especially for the construction and application of predictive models. They assist clinicians in formulating personalized treatment plans tailored to the specific conditions of individual patients. The nomograms we have developed for female lung adenocarcinoma predict patient survival probabilities, facilitating a better evaluation of treatment risks and benefits, thereby supporting more informed decision-making. Specifically, clinicians can use the nomograms to assess 1-year, 3-year, and 5-year survival probabilities, thereby obtaining a clearer understanding of patient prognosis. For early-stage lung adenocarcinoma patients, surgery remains the primary treatment modality. Using the nomograms, clinicians can evaluate patient survival probabilities post-surgery, facilitating more effective communication regarding the associated risks and benefits. Furthermore, for patients with advanced-stage lung adenocarcinoma, the nomograms can assist clinicians in assessing survival probabilities following radiotherapy or chemotherapy, thereby guiding the decision-making regarding combination treatments. The nomograms can also guide follow-up and monitoring plans for patients. For high-risk patients with poor prognosis, more frequent follow-up and more intensive monitoring measures may be necessary to detect and address recurrence or metastasis promptly. Despite the considerable potential benefits of nomograms in clinical practice, some challenges may arise during their implementation. First, clinicians must become familiar with using the nomograms and be able to correctly interpret the predictive results. This may require specialized training and education. Second, the predictive results from the nomograms must be considered alongside the individual circumstances of the patient and should not be entirely relied upon. Clinicians must consider the patient’s overall health status, personal preferences, and other relevant factors. Moreover, the use of nomograms necessitates comprehensive communication between clinicians and patients to ensure patients understand the predictive results and how these results influence treatment decisions.

We constructed nomograms suitable for female lung adenocarcinoma patients using cohorts from the SEER database and an external cohort, both in the absence of systematic studies on the prognosis of this group of patients, and our model also used an external cohort, which reinforces the feasibility of applying these nomograms in different populations. However, this model still has some shortcomings. Firstly, the treatment modalities of lung adenocarcinoma are rapidly developing, and molecular targeted therapy and immune checkpoint inhibitor therapy have been developed to be very mature, and these therapeutic modalities will affect the survival prognosis of the patients, but these data are not available in the SEER database that we used, and the constructed model may be lagging behind the requirements for clinical use. Second, because some key information was missing, we removed a portion of patients during the screening of the data, which may have resulted in selection bias. Finally, the external queues we used were all from the same center, and it is possible that multi-center external validation could provide further evidence of the feasibility of this model for real-world applications. In the future, we hope to apply for the SEER-Medicare database, which will allow us to use patient data that includes information on immunotherapy and targeted therapies to construct predictive models that are more compatible with clinical use.

## 5. Conclusion

This prediction model can accurately predict the prognosis of female lung adenocarcinoma patients, which can provide a certain help for decision-making and prognosis judgment in actual clinical work and provide a certain reference basis for the formulation of female lung adenocarcinoma related guidelines and the development of related clinical trials.

Supplemental digital content “External validation set data “is available for this article (http://links.lww.com/MD/Q351).

## Acknowledgments

We gratefully acknowledge the SEER public databases for providing the data platform, the data contributors, and the developers of the R software and related packages used for retrospective analysis in this study. We also extend our thanks to all those who have contributed to scientific research.

## Author contributions

**Conceptualization:** Feiyang Li.

**Data curation:** Feiyang Li.

**Project administration:** Feiyang Li.

**Software:** Fang Li, Haowei Lu.

**Validation:** Haowei Lu.

**Writing – original draft:** Feiyang Li, Fang Li.

**Writing – review & editing:** Dong Zhao.

## Supplementary Material


