# Supplementary material for: Establishment and validation of prognostic nomograms in female lung adenocarcinoma patients
Source: Medicine (Baltimore). 2025 Oct 17;104(42):e45170. doi: 10.1097/MD.0000000000045170 (PMC12537120; doi:10.1097/MD.0000000000045170)
Supplement: Supplementary file 1 [file medi-104-e45170-s001.docx]

| Variables | Total (n = 130) |
| --- | --- |
|  |  |
| Age, n(%) |  |
| 18-59.9 years | 38 (29.23) |
| 60-79.9 years | 78 (60.00) |
| 80+ years | 14 (10.77) |
| Race, n(%) |  |
| Other | 130 (100.00) |
| Marital status, n(%) |  |
| Married | 127 (97.69) |
| Single | 2 (1.54) |
| Widowed | 1 (0.77) |
| Laterality, n(%) |  |
| Left | 53 (40.77) |
| Right | 77 (59.23) |
| AJCC Stage Group, n(%) |  |
| I | 22 (16.92) |
| II | 9 (6.92) |
| III | 14 (10.77) |
| IV | 85 (65.38) |
| Surgery, n(%) |  |
| No | 91 (70.00) |
| Yes | 39 (30.00) |
| Chemotherapy, n(%) |  |
| No | 64 (49.23) |
| Yes | 66 (50.77) |
| Radiation, n(%) |  |
| No | 95 (73.08) |
| Yes | 35 (26.92) |
| Bone metastasis, n(%) |  |
| No | 79 (60.77) |
| Yes | 51 (39.23) |
| Brain metastasis, n(%) |  |
| No | 101 (77.69) |
| Yes | 29 (22.31) |
| Lung metastasis, n(%) |  |
| No | 100 (76.92) |
| Yes | 30 (23.08) |
| Liver metastasis, n(%) |  |
| No | 115 (88.46) |
| Yes | 15 (11.54) |
|  |  |
